# Migration and child immunization in Nigeria: individual- and community-level contexts

**DOI:** 10.1186/1471-2458-10-116

**Published:** 2010-03-09

**Authors:** Diddy Antai

**Affiliations:** 1 Division of Epidemiology, Institute of Environmental Medicine, Karolinska Institute, Stockholm, Sweden; 2 Division of Global Health & Inequalities, The Angels Trust, Nigeria, Abuja, Nigeria

## Abstract

**Background:**

Vaccine-preventable diseases are responsible for severe rates of morbidity and mortality in Africa. Despite the availability of appropriate vaccines for routine use on infants, vaccine-preventable diseases are highly endemic throughout sub-Saharan Africa. Widespread disparities in the coverage of immunization programmes persist between and within rural and urban areas, regions and communities in Nigeria. This study assessed the individual- and community-level explanatory factors associated with child immunization differentials between migrant and non-migrant groups.

**Methods:**

The proportion of children that received each of the eight vaccines in the routine immunization schedule in Nigeria was estimated. Multilevel multivariable regression analysis was performed using a nationally representative sample of 6029 children from 2735 mothers aged 15-49 years and nested within 365 communities. Odds ratios with 95% confidence intervals were used to express measures of association between the characteristics. Variance partition coefficients and Wald statistic i.e. the ratio of the estimate to its standard error were used to express measures of variation.

**Results:**

Individual- and community contexts are strongly associated with the likelihood of receiving full immunization among migrant groups. The likelihood of full immunization was higher for children of rural non-migrant mothers compared to children of rural-urban migrant mothers. Findings provide support for the traditional migration perspectives, and show that individual-level characteristics, such as, migrant disruption (migration itself), selectivity (demographic and socio-economic characteristics), and adaptation (health care utilization), as well as community-level characteristics (region of residence, and proportion of mothers who had hospital delivery) are important in explaining the differentials in full immunization among the children.

**Conclusion:**

Migration is an important determinant of child immunization uptake. This study stresses the need for community-level efforts at increasing female education, measures aimed at alleviating poverty for residents in urban and remote rural areas, and improving the equitable distribution of maternal and child health services.

## Background

In spite of significant reductions in child mortality in developing countries in recent decades, more than 10 million children younger than 5 years continue to die yearly [1,2]. Vaccine-preventable diseases (VPDs) constitute major causes of morbidity and mortality in Africa. About 95 percent of the estimated 14 million deaths of children below 5 years of age worldwide occur in developing countries; approximately 70 percent of these deaths are due to vaccine-preventable diseases [3]. In Nigeria, over one million children die annually from preventable diseases, making the country one of the least successful of African countries in achieving improvements in child survival during the past four decades [4]. For instance, in 2005, an estimated 25% of one-year-old children had received the third dose of diphtheria, pertussis and tetanus (DPT) vaccine [5].

Widespread disparities in immunization coverage persist between and within regions in Nigeria to the disadvantage of children of parents in the lowest socio-economic quintiles, with no education, and residing in rural areas (especially in the north) [6]. The rich geographical, cultural, ethnic, and socio-economic diversity of Nigeria implies that immunization uptake varies between and within geographical regions. These variations may subsequently lead to clusters of children that are under-vaccinated, thereby increasing the vulnerability of the rest of the population to major outbreaks of vaccine-preventable diseases. Childhood immunization uptake remains critical in Nigeria despite sporadic success in the reduction of VPD in Nigeria [7].

Vaccines are among the most effective preventive health measures in reducing child mortality, morbidity and disability [8,9]. The introduction of appropriate vaccines for routine use on infants has resulted in drastic reductions in vaccine-preventable diseases [2,10]. In spite of this, the six diseases (measles, pertussis, diphtheria, tuberculosis, tetanus, and poliomyelitis) included in the expanded program of immunization (EPI) continue to cause serious morbidity and mortality in Africa in general [11]. Identifying groups with low immunization uptake, and the behavioural processes associated with low immunization uptake are important for the success of immunization campaigns, as well as the efficient allocation of public health resources [12].

Migration has been implicated as one of the behavioural processes influencing low immunization uptake [13]. Population migration is a choice process that is influenced by socio-economic, demographic and cultural factors [14]. Socio-economic factors, such as the expectation of better earnings and employment opportunities, access to modern amenities, seem to a greater extent instrumental in the motives of the rural residents to migrate into cities [14]. Several studies have documented health differentials by rural-urban place of residence in developing countries [15,16]. However, not much is known about changes in these differentials over time as levels of rural-urban migration increases and as the socio-economic development processes unfold. For instance, urban-rural mortality gap has narrowed in Kenya within the last fifty years due to rapidly declining rural mortality over most of that period. In more recent times, the urban-rural mortality gap has resulted primarily from a stalling, and even upturn in urban mortality, due to the deterioration of living conditions in rapidly growing cities [16,17]. With the increased rural-urban migration in most sub-Saharan countries including Nigeria, migration is a likely determinant of immunization uptake. However, little is known about the role of population redistribution on child health outcomes such as childhood immunization [13].

### Theoretical framework

Three perspectives of migration could be used to explain the disparities in child immunization uptake among migrant groups. These include: *i) *disruption; *ii) *selection; and *iii) *adaptation. *Migrant disruption *posits that the process of migration disrupts the natural progression of demographic events in the lives of the migrants, such as break in mothers' network of social- and financial support, as well as social & cultural practices [18]. This disruption may interfere with child immunization uptake and consequently necessitate a significant period of adaptation, and is associated with the migration process itself. Migrant status variable was used to test the migrant disruption perspective in this study.

*Migrant selectivity *suggests that migration is selective for people with characteristics that are favourable for child health outcomes such as education, occupation and wealth index. According to this perspective, an observed increase in the risks of immunization uptake for children of rural-urban migrants is thought to be mainly associated with the migrant characteristics that also increase their propensity to migrate [18-20]. In this study, demographic and socio-economic characteristics were used to operationalize migrant selectivity perspective.

*Migrant adaptation *posits that differential health outcomes (in this case, immunization uptake) among the children of rural-urban migrants and non-migrants are associated with the difficulty of migrants to adjust to, and effectively use services and facilities in the new urban environment [21]. Migrant adaptation perspective in this study was operationalized by characteristics associated with health care utilization.

The role played by individual- and community-level characteristics in migrant adaptation and full immunization uptake as they move between social settings has important policy implications, both for health outcomes in Nigeria and other developing countries undergoing significant internal migration.

The aim of this study was therefore to examine the effects of individual- and community-level characteristics of migrant groups on the likelihood of the full immunization uptake of their children.

## Methods

Data from the 2003 Nigeria Demographic and Health Survey (DHS) was used in this study. This is a nationally-representative probability sample, collected using a stratified two-stage cluster sampling procedure. A full report and detailed description of the data collection procedures are presented elsewhere [22]. Birth history data, such as, sex, month and year of birth, survivorship status and current age or, if the child had died, ages at death were also collected for each of these births. Immunization status of a child was determined from vaccination cards shown to the DHS interviewer. In the absence of vaccination cards, mothers were asked to recall whether theirchild had received BCG, polio, DPT (including the number of doses for each) and measles vaccinations.

### Measures

#### Outcome

The outcome variable is the risk of a child 12 months of age and older receiving full immunization (i.e. all of the eight required vaccinations in the EPI programme).

Routine immunization schedule in Nigeria stipulates that infants should be vaccinated with the following vaccines: a dose of Bacillus Calmette-Guerin (BCG) vaccine at birth (or as soon as possible); three doses of diphtheria, pertussis and tetanus (DPT) vaccine at 6, 10 and 14 weeks of age; at least three doses of oral polio vaccine (OPV) - at birth, and at 6, 10 and 14 weeks of age; and one dose of measles vaccine at 9 months of age [23,24]. A child was considered to have received full immunization status when they have received the full complement of eight vaccinations according to the EPI programme mentioned above.

#### Exposures

##### Migrant status

Migrant status was categorized as: urban non-migrant, rural non-migrant and rural-urban migrant.

A migrant was defined as a person who moved between any combination of rural and urban areas in the 10 years prior to the survey. Migration histories are not routinely collected in the Demographic and Health Surveys; however, basic information relating to number of years spent in the respondents current place of residence are collected, as well as place of residence (previous and current). These were used to establish migration status and to identify four migration streams: urban-to-urban, rural-to-rural, rural-to-urban and urban-to-rural. A variable that categorized the migration streams into rural-to-urban migrants, rural non-migrants, and urban non-migrants was created. Migrants in the rural-to-rural and urban-to-urban streams made up the rural- and urban non-migrants, while urban-to-rural migrants were excluded from the analysis. Migration status of a person was defined by a person changing their place of residence across an administrative boundary. Visitors were excluded from the analysis. For instance, a woman who reported previous residence as rural and current residence as urban was classified as a rural-urban migrant. The non-migrant groups are classified as rural- or urban non-migrant depending upon their reported duration at the place of residence as "always".

##### Individual-level explanatory factors

A number of child- and mother-level characteristics may potentially confound the relationship between migration status and likelihood of full immunization among children younger than 5 years of age.

*Demographic *characteristics assessed included: as: *a) *birth order/birth interval, created by merging "birth order" and the "preceding birth interval" into one variable. The variable 'preceding birth interval' is the interval before the birth of the child in question. As such, the effect of the preceding birth interval is considered in relation to the younger of the two children. Ideally, first births are left out of the analysis of preceding birth interval and survival of the preceding child because they are not preceded by another birth. In order to enable the inclusion of first births in the analysis, first births in this study were merged with those with a preceding birth interval of 24 months or longer. This merged variable was classified into seven categories as: first births, birth order 2-4 with short birth interval (< 24 months), birth order 2-4 with medium birth interval (24-47 months), birth order 2-4 with long birth interval (48+ months), birth order 5+ with short birth interval (< 24 months), birth order 5+ with medium birth interval (24-47 months), and birth order 5+ with long birth interval (48 months); *b) *sex of the child, categorized as: male and female; *c) *mother's age, grouped as: 15-18, 19-23, 24-28, 29-33, and 34 years and older; *d) *mother's age at birth of first child, categorized as: 18 years or less and 19 years or older; and *e) *marital status, categorized as: single, married and formerly married.

*Socio-economic *characteristics were assessed as: *a) *mothers' education, categorized as: no education, primary, and secondary or higher education; *b) *mother's occupation, grouped as: professional/technical/managerial; clerical/sales/services/skilled manual; agricultural self employed/agricultural employee/household & domestic/unskilled manual occupations; and not working; and *c) *wealth index, which is used in the absence reliable data on incomes and expenditures in the demographic and health survey. This is a composite index and indicator of the socio-economic status of households that assigns weights or factor scores generated by principal component analysis to information on household assets collected from censuses and surveys. Household socio-economic indicators included those relating to household ownership of durable assets and household environmental conditions; these were used to compute the index. Principal components analysis allows each asset owned to be given a score and the factor loading scores used to create linear composites of each household socio-economic status variable. The socio-economic index generated is subsequently divided into quintiles of socio-economic status, categorized as: poorest, poorer, middle, richer and richest.

*Health care utilization *was assessed as: *a) *mother received tetanus toxoid injections in pregnancy, categorized as: yes and no; *b) *place of delivery of child, categorized as: home, and hospital facility; and *c) *prenatal care by doctor, categorized as: yes and no.

##### Community-level explanatory factors

These included: *a) *mothers' region of residence, categorized according to the six geo-political zones in Nigeria, as: North Central, North East, North West, South East, South South, and South West; and *b) *three contextual variables, which were at the level of the primary sampling unit (PSU) (*n *= 365) were used.; *i) *community prenatal care by doctor, defined as the percentage of mothers who received prenatal care by a doctor during pregnancy within the PSU, and categorized as: low, and high; *ii) *community hospital delivery, defined as the percentage of mothers who delivered their child in a hospital facility within the PSU, and categorized as: low, middle, and high. Prenatal care directly increases the chances that the mother would subsequently access health care services for her child, such as institutional delivery and immunization [25,26]. Thus, the proportion of mothers that delivered in a hospital setting is a predictor of child immunization uptake. Hospital delivery is one of the most important preventive measures against maternal and child health outcomes, and an important determinant of full immunization [27,28]; and *iii) *community mother's education, defined as the percentage of mothers with secondary or higher education within the PSU, and categorized as: low and high. Higher levels of maternal education are associated with better child health outcomes, such as child immunization rates [29,30].

PSUs or clusters are administratively-defined areas used as proxies for "neighbourhoods" or "communities" [31]. They are small and designed to be fairly homogenous units with respect to population socio-demographic characteristics, economic status and living conditions, and are made up of one or more enumeration areas (EAs), which are the smallest geographic units for which census data are available in Nigeria. Each cluster consisted of a minimum of 50 households, with a contiguous EA being added when a cluster had less than 50 households [22].

The simultaneous inclusion of both individual- and neighbourhood-level predictors in regression equations with individuals as the units of analysis, permits: *i) *the examination of neighbourhood or area effects after individual-level confounders have been controlled; *ii) *the examination of individual-level characteristics as modifiers of the area effect (and vice versa); and *iii) *the simultaneous examination of within- and between neighbourhood variability in outcomes, and of the extent to which between-neighbourhood variation is "explained" by individual- and neighbourhood-level characteristics [31,32].

### Statistical analysis

#### Characteristics of the study population

The distribution of the children and mothers in the sample was assessed by migration status and socio-economic characteristics. Normalized sample weights provided in the DHS data were used for all analyses in order to adjust for non-response and enable generalization of findings to the general population. These analyses were done using Stata 10 [33].

#### Multilevel logistic regression modelling

A three-level multilevel logistic regression model to account for the hierarchical structure of the DHS data [34] was used. Children (level 1), were nested within mothers (level 2), who were in turn nested within communities (level 3). Five models were fitted containing variables of interest, grouped into categories. **Model 1 **contained only mother's migration status as the only exposure variable. **Model 2 **included migration status and demographic characteristics of children and mothers (sex of the child, birth order/birth interval, mother's age and mother's age at birth of first child). **Model 3 **contained migration status and socio-economic variables (mother's education, mother's occupation and wealth index), and **Model 4 **contained migration status and health care utilization (mother received tetanus toxoid injections in pregnancy, place of delivery of child and prenatal care by doctor). Finally, **Model 5 **contained community-level variables (mother's region of residence, community prenatal care by doctor, community hospital delivery, and community mother's education). In each of the five models, migration status was fitted with a different category of exposure variables against the risk of full immunization. This modelling strategy is intended to enable a comparison of the influence of each of the different exposure variables on the association between migration and the likelihood of full immunization.

#### Measures of association (fixed effects)

The association between the likelihood of full immunization and migration status were expressed as odds ratio (OR) and 95% confidence intervals (95% CIs).

#### Measures of variation (random effects)

The random effects (measures of variation) were expressed as Variance Partition Coefficient (VPC) and proportional change in variance (PCV). The variance partition coefficient (VPC) measures the extent that siblings resemble each other more than they resemble children from other families in relation to the likelihood of full immunization. A large VPC value (close to 1) indicates maximally segregated clusters, and a low VPC value (close to zero) suggests homogeneous risk of under-five mortality among clusters. Statistical testing of the population variance was performed using the Wald statistic i.e. the ratio of the estimate to its standard error [35]. The multilevel analyses were performed using MLwiN software package 2.0.2 [36], with Binomial, Penalized Quasi-Likelihood (PQL) procedures [37]. Random slope variance indicates whether contextual phenomenon differs in magnitude for different groups of people and whether the community level modifies associations between individual-level exposures.

### Ethical considerations

This study is based on analysis of secondary data with all participant identifiers removed. The survey was approved by the National Ethics Committee in the Federal Ministry of Health, Nigeria and the Ethics Committee of the Opinion Research Corporation Macro International, Incorporated (ORC Macro Inc.), Calverton, USA. Informed consent was obtained from the participants prior to participation in the survey, and data collection was done confidentially. Permission to use the DHS data in this study was obtained from ORC Macro Inc.

## Results

### Characteristics of the study population (Table 1)

**Table 1:** Number and proportion of children in by vaccination type and migration status of the mother

Vaccination	Urban non-migrant N (%)	Rural non-migrant N (%)	Rural-urban migrant N (%)	Total N (%)
**BCG**				
No	355 (41.4)	274 (26.9)	1123 (69.9)	1752 (50.3)
Yes	502 (58.6)	746 (73.1)	484 (30.1)	1732 (49.7)
**DPT 1**				
No	453 (52.9)	378 (37.2)	1214 (75.6)	2045 (58.8)
Yes	404 (47.1)	638 (62.8)	391 (24.4)	1433 (41.2)
**OPV 1**				
No	322 (37.7)	227 (22.3)	651 (40.6)	1200 (34.5)
Yes	532 (62.3)	792 (77.7)	954 (59.4)	2278 (65.5)
**DPT 2**				
No	547 (63.9)	465 (45.9)	1294 (80.9)	2306 (66.4)
Yes	309 (36.1)	549 (54.1)	306 (19.1)	1164 (33.6)
**OPV 2**				
No	429 (50.7)	324 (32.0)	833 (52.2)	1586 (45.9)
Yes	417 (49.3)	689 (68.0)	763 (47.8)	1869 (54.1)
**DPT 3**				
No	627 (73.2)	595 (58.7)	1413 (88.3)	2635 (75.9)
Yes	229 (26.8)	419 (41.3)	187 (11.7)	835 (24.1)
**OPV 3**				
No	603 (71.3)	586 (57.8)	1169 (73.2)	2358 (68.2)
Yes	243 (28.7)	427 (42.2)	427 (26.8)	1097 (31.8)
**Measles**				
No	535 (62.8)	472 (46.4)	1266 (79.4)	2273 (65.6)
Yes	317 (37.2)	545 (53.6)	329 (20.6)	1191 (34.4)
**Full immunization**				
No	503 (84.8)	457 (75.7)	1192 (91.5)	2152 (86.1)
Yes	90 (15.2)	147 (24.3)	111 (8.5)	348 (13.9)

A higher proportion of urban non-migrant children had received BCG (58.6%) and OPV 1 (62.3%). Most of the rural non-migrant children had received BCG (73.1%), DPT 1 (62.8%), DPT 2 (54.1%), OPV 1 (77.7%), OPV 2 (68.0%) and Measles (53.6%) vaccines. With the exception of OPV 1, most of the rural-urban migrant children had not received the rest of the vaccines in the programme. Most children had not been fully immunized, as only 8.5% of the rural-urban migrant children had been fully immunized. Rural non-migrant (24.3%) children had the highest levels of full immunization amongst children from the three migrant groups. Urban non-migrant (15.2%) had slightly higher levels of full immunization than children of rural-urban migrants.

Exposure variables included in the multilevel analysis are presented in Table 2.

**Table 2:** Exposure variables used in modelling the association between migration status and the likelihood of full immunization

Model 1 Migration status	Model 2 Demographic	Model 3 Socioeconomic	Model 4 Health care utilization	Model 5 Community
Migration status	Migration status	Migration status	Migration status	Migration status
	Birth order/birth interval of child	Mother's education	Mother received tetanus toxoid injections in pregnancy	Mother's region of residence
	Sex of child	Mother's occupation	Place of delivery	Community prenatal care by doctors
	Mother's age	Wealth index	Mother received prenatal care by doctor	Community hospital delivery
	Mother's age at birth of first child			Community mother's education
	Mother's marital status			

#### Measures of association (fixed effects) (Table 3)

**Table 3:** Measures of association (fixed effects i.e. odds ratios and 95% confidence intervals for multivariable multilevel logistic regression models)

Characteristics	Model 1 (Migration status)	Model 2 (Demographic)	Model 3 (Socio-economic)	Model 4 (Health care utilization)	Model 5 (Community)
	**OR (95% CI)**	**OR (95% CI)**	**OR (95% CI)**		**OR (95% CI)**

*Migration status*					
Rural-urban migrant	1	1	1		1
Rural non-migrant	2.36 (1.75 - 3.19)*	2.03 (1.50 - 2.75)*	1.43 (1.01 - 2.05)**	0.62 (0.43 -0.89)**	1.89 (1.23 - 2.90)*
Urban non-migrant	1.67 (1.20 - 2.32)*	1.54 (1.11 - 2.15)*	1.20 (0.95 - 1.51)	1.13 (0.77 - 1.66)	1.35 (0.88 - 2.07)
*Birth order/birth interval*					
First birth (order 1)		1.16 (0.80 - 1.69)			
Order 2-4 & <24 months		1.13 (0.75 - 1.70)			
2-4 & medium bi 24-47 months		0.77 (0.47 - 1.26)			
Order 2 -4 & 48+ months		1			
Order 5+ & < 24 months		0.59 (0.33 - 1.05)			
Order 5+ & 24- 47 months		0.64 (0.41 - 0.97)*			
Order 5+ & 48+ months		0.98 (0.57 - 1.67)			
*Sex of child*					
Female		1.03 (0.82 - 1.30)			
Male		1			
*Mother's age*					
15 - 18		0.50 (0.22 - 1.12)			
19 - 23		0.81 (0.54 - 1.21)			
24 - 28		1			
29 - 33		1.08 (0.75 - 1.55)			
≥ 34		1.54 (1.03 - 2.30)*			
*Mother's age at birth of first child*					
18 years or less		0.74 (0.56 - 0.99)**			
19+		1			
*Marital status*					
Single		1.29 (0.53 - 3.15)			
Divorced		1			
Married		0.85 (0.49 - 1.47)			
*Mothers' education*					
No education			0.72 (0.50 - 1.03)		
Primary			0.97 (0.69 - 1.36)		
Secondary or higher			1		
*Mothers' occupation*					
Not working			0.57 (0.34 - 0.94)*		
Agricultural self-employed/agric- ultural employee/household & domestic/unskilled manual occupations			0.82 (0.45 - 1.48)		
Clerical/sales/services/skilled manual			0.57 (0.35 - 0.93)*		
Professional/Technical/Management			1		
*Wealth index*					
Poorest			0.45 (0.26 - 0.77)		
Poorer			0.43 (0.26 - 0.70)*		
Middle			0.62 (0.41 - 0.95)*		
Richer			0.54 (0.37 - 0.78)*		
Richest			1		
*Received tetanus toxoid injection during pregnancy*					
No				0.49 (0.33 - 0.71)*	
Yes				1	
*Place of delivery*					
Home				0.67 (0.48 - 0.93)**	
Hospital facility				1	
*Received prenatal care by doctor*					
No				0.82 (0.59 - 1.13)	
Yes				1	
*Region of residence*					
North Central					1.11 (0.67 - 1.85)
North East					0.57 (0.30 - 1.11)
North West					0.51 (0.25 - 1.01)
South East					0.61 (0.32 - 1.14)
South South					0.48 (0.26 - 0.92)
South West					1
*Community prenatal care by doctor*					
Low					1.37 (0.79 - 2.39)
High					1
*Community hospital delivery*					
Low					0.48 (0.28 - 0.82)*
Middle					1
High					1.43 (0.88 - 2.33)
*Community mothers' education*					
*Low*					0.76 (0.51 - 1.12)
*Middle*					1
*High*					0.89 (0.60 - 1.32)

A total of 6029 children were nested within 3725 mothers who were in turn nested within 365 communities. The sequence of entry of the variables used in the multilevel model is presented in Table 2.

The association between migration status and the likelihood of full immunization is presented in Table 3. On fitting migration status into **Model 1**, the likelihood of full immunization for children of rural non-migrant mothers was more than two times (OR = 2.36, 95% CI = 1.75 - 3.19) that for children of rural-urban migrant mothers. Children of urban non-migrant mothers had 67% higher likelihood (OR = 1.67, 95% CI = 1.20 - 2.32) of full immunization compared to children of rural-urban migrant mothers. This indicates that mothers' migration significantly influenced the likelihood of their child receiving full immunization.

Demographic characteristics (birth order/birth interval, sex of the child, mother's age, mother's age at birth of first child and marital status) were adjusted for in **Model 2**. This slightly attenuated the association between rural non-migrant (OR = 2.03, 95% CI = 1.50 - 2.75) and urban non-migrant (OR = 1.54, 95% CI = 1.11 - 2.15) children in the likelihood of full immunization. This indicates that the effect of migration on full immunization is independent of demographic characteristics. In addition, the likelihood of full immunization was significantly lower for children of 5+ birth order after medium birth interval 24 - 47 months (OR = 0.64, 95% CI 0.41 - 0.97) and for children whose mothers who gave birth to their first child at 18 years or less (OR = 0.74, 95% CI 0.56 - 0.99). In contrast, the likelihood of full immunization was significantly higher for children of mothers 34 years or older (OR = 1.54, 95% CI 1.03 - 2.30).

Socio-economic characteristics (mother's education, mother's occupation, and wealth index) were introduced along with migration status in **Model 3**. This further attenuated the association between children of rural non-migrants (OR = 1.43, 95% CI = 1.01 - 2.05) in the likelihood of full immunization, while the likelihood of full immunization for children of urban non-migrants became non-significant. This suggests that differences in the likelihood of full immunization were explained to a greater extent by the differences in the distribution of socio-economic characteristics of the migrant and non-migrant groups. In addition, children of mothers without employment (OR = 0.57, 95% CI = 0.34 - 0.94), clerical, sales, services, skilled manual employees (OR = 0.57, 95% CI = 0.35 - 0.93) had higher likelihood of full immunization compared to children of professional, technical, management workers, respectively. Children of mothers in the poorest (OR = 0.45, 95% CI = 0.27 - 0.77), poorer (OR = 0.43, 95% CI = 0.26 - 0.70), middle (OR = 0.62, 95% CI = 0.41 - 0.95), and richer (OR = 0.54, 95% CI = 0.37 - 0.78) wealth quintiles had lower likelihood of full immunization compared to children of mothers in the richest wealth quintile.

**Model 4 **adjusted for characteristics associated with health care utilization (mother received tetanus toxoid injections in pregnancy, place of delivery, and prenatal care by doctor) along with migration status. This further attenuated the effect of the association between full immunization and migration status, as the likelihood of full immunization was 38% lower for children of rural non-migrants (OR = 0.62, 95% CI = 0.43 - 0.89) compared to children of rural-urban migrants. This means that differences in full immunization between the migrant and non-migrant groups could be partly explained by the unequal utilization of health care services between these different groups. In addition, the likelihood of full immunization was 51% and 33% lower for children of mothers who had not received tetanus injection during pregnancy (OR = 0.49, 95% CI = 0.33 - 0.71), and children of mothers who delivered at home (OR = 0.49, 95% CI = 0.33 - 0.71), compared with children of mothers who received tetanus injection during pregnancy and those delivered in a hospital facility, respectively.

Finally, **Model 5 **adjusted for community-level variables (mother's region of residence, community prenatal care by doctor, community hospital delivery, and community mother's education) in order to assess the effects of community-level and regional variations in the provision of services on the risk of full immunization. The likelihood of full immunization was 89% higher for children of rural non-migrants (OR = 1.89, 95% CI = 1.23 - 2.90) compared to children of rural-urban migrants. This means that full immunization differentials between the migrant and non-migrant groups is independent of the community-level characteristics.

#### Measures of variation (random effects) (Table 4)

**Table 4:** Measures of variation (random effects)

	**Model 1**^a^	**Model 2**^b^	**Model 3**^c^	**Model 4**^d^	**Model 5**^e^
**Random effects**					
*Community-level*					
Variance (SE)	0.595(0.160)*	0.494 (0.158)*	0.432 (0.157)*	0.317 (0.174)*	0.627 (0.195)*
VPC (%)	13.6	11.3	9.9	8.8	14.5
*Mother-level*					
Variance (SE)	0.486 (0.247)*	0.574 (0.255)*	0.634 (0.258)*	0.000 (0.190)	0.417 (0.280)*
VPC (%)	11.1	13.2	14.5	0	9.6

^a ^Model 1: contained only migration status;

^b ^Model 2: migration status and demographic characteristics;

^c ^Model 3: migration status and socio-economic characteristics;

^d ^Model 4: migration status and characteristics indicating health care utilization;

^e ^Model 5: migration status and community-level characteristics.

VPC = Variance partition coefficient; SE = Standard error: OR = Odds ratio; CI = Confidence Interval.

*p < .05;

**p < .01;

***p < .001

The variance was significant across mothers (τ = 0.486, *p *< 0.5) and communities (τ = 0.595, *p *< 0.5) on fitting migration status into **Model 1**. The proportional change in variance (PCV) indicates that 11.1% and 13.6% of the variance in the likelihood of full immunization across mothers and communities, respectively, were explained by migration status of the population. The variance remained significant across mothers (τ = 0.574, *p *< 0.5) and communities (τ = 0.494, *p *< 0.5) after adjusting for demographic characteristics in **Model 2**. As indicated by the PCV, 13.2% and 11.3% of the variance in the likelihood of full immunization across mothers and communities, respectively, were explained by demographic characteristics. After adjusting for socio-economic characteristics in **Model 3**, the variance across mothers (τ = 0.634, *p *< 0.5) and communities (τ = 0.432, *p *< 0.5) remained significant. As indicated by the PCV, 14.5% of the variance in the likelihood of full immunization across mothers, and 9.9% of the variance in the likelihood of full immunization across communities were explained by socio-economic characteristics.

Only the variance across communities (τ = 0.317, *p *< 0.5) remained statistically significant after adjusting for characteristics associated with health care utilization in **Model 4**. The PCV indicates that 8.8% of the variance in the likelihood of full immunization across communities was also explained by characteristics associated with health care utilization. The variance of 0% across mothers indicates that mothers are similar with respect to the likelihood of full immunization, and are therefore irrelevant in understanding the variation in full immunization after adjusting for characteristics associated with health care utilization. As indicated by the proportional change in variance (PCV), 25.7% and 1% of the variance in the risks of under-five mortality across mothers and communities, respectively, were explained by the individual-level socio-economic characteristics. Finally, in **Model 5**, the variance was significant across mothers (τ = 0.417, *p *< 0.5) and across communities (τ = 0.627, *p *< 0.5) after adjusting for community-level characteristics. As indicated by the PCV, 9.6% and 14.5% of the variance in the likelihood of full immunization across mothers and communities, respectively, were explained by community-level characteristics.

## Discussion

Having taken the hierarchical nature of multilevel analysis into consideration, this study demonstrated that the individual- and community contexts were strongly associated with the likelihood of receiving full immunization among migrant groups. This study found a strong correlation between migration status of the mothers (rural non-migrant) and the likelihood of their child receiving full immunization. This is an expected finding, considering that as geographical and social mobility lead to rapid urbanization, changes in social and economic opportunities alter people's lifestyle and health outcomes. Thus, internal migration produces a range of risks and opportunities, which in the case of many developing countries leans towards risk for the poor in both non-migrant and migrant groups. Despite generally improvement in services (e.g. maternal and child health services) within urban areas, the supposed urban advantage is seemingly offset by the cost of these services, migrants' lack of social networks, and the poor state of the economy. Consequently, poor rural-urban migrants and urban non-migrants cannot afford to utlilize these improved urban services. As rural-urban migration disrupts individuals' income-generation ability, access to health care services, as well as family and community attachments, it could be concluded that the likelihood of full immunization for children of rural-urban migrants is associated with the disruption caused by the migration itself (*migrant disruption*). Similar result has been reported in previous studies [13,38].

Migration explains only part of the variance in the likelihood of full immunization. Several other explanatory characteristics also help in explaining the resulting immunization differentials in this study. Demographic and socio-economic characteristics significantly attenuated the risks of full immunization. Among the demographic characteristics, children were less likely to be fully immunized if they were of 5+ birth order after medium birth interval 24 - 47 months. This was probably associated with mothers being unable to cater adequately to the health needs of many children or even negligence [39]. Similar findings have been reported in other studies [40,41]. Age of mothers at birth of first child (18 years or less) was significantly associated with reduced likelihood of full immunization. Possible explanations include lack of money for transport, and lack of support offered by the mothers' social network regarding advice, information, counselling, material and emotional support, as well as health education. This is consistent with findings from other studies [42,43]. In contrast, having a mother 34 years or older increased the likelihood of a child being fully immunized. This may be associated with increased maturity, awareness, and social network of older mothers. In support of this, the present study found evidence of socio-economic disparities in the likelihood of full immunization among migrant groups. Occupation of the mothers (unemployment, and clerical/sales/services/skilled manual workers), and lower wealth status were significantly associated with lower risks of full immunization. Lower status occupations and lower wealth status may be an obstacle to full immunization because of difficulty in taking time away from work, as opposed to a more supportive workplace commonly associated with occupations of higher status. Similar effect of lower status occupations, though relating to breastfeeding initiation, has been reported [44]. Demographic and socio-economic characteristics are therefore important in explaining the full immunization differentials found between migrant and non-migrant groups, and indicate that *migrant selectivity *is a significant factor in the immunization of children of migrants.

Health care utilization also significantly attenuated the likelihood of full immunization, and explained the differentials in full immunization between children of migrants and non-migrants. The likelihood of full immunization was lower for children of mothers that did not receive tetanus toxoid injection during pregnancy, and for children of mothers who delivered at home, compared to children of mothers who did receive tetanus toxoid and delivered in a hospital facility, respectively. This is consistent with prior studies, which showed that prenatal care increases the chances that mothers would subsequently access health care services for their child, such as hospital delivery and immunization [45,46]. Disparities in health care utilization may partly explain differentials in full immunization between migrants and non-migrants. It could be argued that rural-urban migrants may have failed to adapt fully into their new urban environment due to socio-economic disadvantage and lack of social support, indicating that *migrant adaptation *is a significant factor in the full immunization uptake amongst migrant groups.

Results of this study reveal significant variability in the likelihood of full immunization across communities. The likelihood of full immunization was lower for children living in the South South region (also known as the Niger Delta) where vaccine-preventable deaths are responsible for 20% infant mortality [47]. This is consistent with other reports which associate reduced risks of immunization with inadequate health care facilities and services, poverty, and inaccessibility of the regions where these children reside [48,49]. Living in communities with low proportion of mothers who had hospital delivery was associated with lower risks of full immunization compared to living in a community with high proportion of hospital delivery. This is an expected finding, given that community health services have been shown to be important correlates of health outcomes in developing countries [45,46], in that mothers who deliver at home are generally more likely to be non-users of health services. This provides an explanation for full immunization differentials between children of migrant and non-migrant mothers over and above the individual characteristics of the mother or child. Significant variance left at the community- and individual levels suggests that more research on community and individual factors among migrant groups is necessary, such as other determinants of child survival not included in this study.

### Strengths and Limitations

Findings in this study should be considered in light of the following limitations. First, other factors not addressed in the present study are also likely to be important determinants of full immunization among migrant and non-migrant groups. Second, DHS surveys do not collect data on household income or expenditure, which are the indicators commonly used to measure wealth. The assets-based wealth index used here is only a proxy indicator for household economic status, which may not always produce results similar to those obtained from direct measurements of income and expenditure where such data are available or can be collected reliably [50]. Third, the administratively defined boundaries used as a proxy for neighbourhoods in this study may non-differentially misclassify individuals into an inappropriate administrative boundary, which can generate information biases and reduce the validity of analyses. Fourth, other community correlates likely to affect the likelihood of full immunization were not included in the analysis. Some of these include variables not measured or not measurable, such as distance to immunization centres, and quality of immunization services. Fifth, DHS data did not contain direct information about the social networks of the migrant groups. Hence, the extent of the mothers' social networks in the community they reside in could not be assessed.

The strengths are worthy of mention. First, the novelty of this study beyond previous research on the inter-relationship of migration and child immunization is its use of multilevel modelling to test the theoretical perspectives of migration on the likelihood of full immunization. Second, the DHS surveys are nationally-representative and allow for generalization of the results across the country [51]. Third, variables in the DHS surveys are defined similarly across countries and results are therefore comparable across countries [52].

## Conclusion

This study showed that individual- and community-level characteristics are important determinants of the likelihood of full immunization uptake among migrant groups. That the likelihood of full immunization was higher for children of rural non-migrant mothers as opposed to children of rural-urban migrants is indicative of alterations in health outcomes of rural-urban migrants. This emphasizes the need for enhanced community-level measures in urban communities that would enhance improved full immunization uptake, such as increased female education, increased community health campaigns targeting mothers who deliver at home, and a general improvement of the socio-economic situation of people in urban communities. There is also a need for improvement in the quality and equitable spatial distribution of maternal and child health services. The fact that high birth order, home delivery and low proportion of mothers who delivered in hospital were independent predictors of reduced likelihood of full immunization uptake stresses the need to broaden child survival measures to include mothers at pre-pregnancy and pregnancy stages.

## Competing interests

The authors declare that they have no competing interests.

## Pre-publication history

The pre-publication history for this paper can be accessed here:

http://www.biomedcentral.com/1471-2458/10/116/prepub
